# Asporin Is a Fibroblast-Derived TGF-β1 Inhibitor and a Tumor Suppressor Associated with Good Prognosis in Breast Cancer

**DOI:** 10.1371/journal.pmed.1001871

**Published:** 2015-09-01

**Authors:** Pamela Maris, Arnaud Blomme, Ana Perez Palacios, Brunella Costanza, Akeila Bellahcène, Elettra Bianchi, Stephanie Gofflot, Pierre Drion, Giovanna Elvi Trombino, Emmanuel Di Valentin, Pino G. Cusumano, Sylvie Maweja, Guy Jerusalem, Philippe Delvenne, Eric Lifrange, Vincent Castronovo, Andrei Turtoi

**Affiliations:** 1 Metastasis Research Laboratory, GIGA–Cancer, University of Liège, Liège, Belgium; 2 Department of Pathology, University Hospital Liège, University of Liège, Liège, Belgium; 3 Biotheque, University of Liège, Liège, Belgium; 4 Animal Facility, GIGA–Cardiovascular Sciences, University of Liège, Liège, Belgium; 5 Department of Pharmacy and Health and Nutritional Sciences, University of Calabria, Arcavacata di Rende, Cosenza, Italy; 6 GIGA–Viral Vectors Platform, University of Liège, Liège, Belgium; 7 Department of Senology, University Hospital Liège, University of Liège, Liège, Belgium; 8 Department of Abdominal Surgery, University of Liège, Liège, Belgium; 9 Department of Medical Oncology, University Hospital Liège, University of Liège, Liège, Belgium; Fred Hutchinson Cancer Research Center, UNITED STATES

## Abstract

**Background:**

Breast cancer is a leading malignancy affecting the female population worldwide. Most morbidity is caused by metastases that remain incurable to date. TGF-β1 has been identified as a key driving force behind metastatic breast cancer, with promising therapeutic implications.

**Methods and Findings:**

Employing immunohistochemistry (IHC) analysis, we report, to our knowledge for the first time, that asporin is overexpressed in the stroma of most human breast cancers and is not expressed in normal breast tissue. In vitro, asporin is secreted by breast fibroblasts upon exposure to conditioned medium from some but not all human breast cancer cells. While hormone receptor (HR) positive cells cause strong asporin expression, triple-negative breast cancer (TNBC) cells suppress it. Further, our findings show that soluble IL-1β, secreted by TNBC cells, is responsible for inhibiting asporin in normal and cancer-associated fibroblasts. Using recombinant protein, as well as a synthetic peptide fragment, we demonstrate the ability of asporin to inhibit TGF-β1-mediated SMAD2 phosphorylation, epithelial to mesenchymal transition, and stemness in breast cancer cells. In two in vivo murine models of TNBC, we observed that tumors expressing asporin exhibit significantly reduced growth (2-fold; *p* = 0.01) and metastatic properties (3-fold; *p* = 0.045). A retrospective IHC study performed on human breast carcinoma (*n* = 180) demonstrates that asporin expression is lowest in TNBC and HER2+ tumors, while HR+ tumors have significantly higher asporin expression (4-fold; *p* = 0.001). Assessment of asporin expression and patient outcome (*n* = 60; 10-y follow-up) shows that low protein levels in the primary breast lesion significantly delineate patients with bad outcome regardless of the tumor HR status (area under the curve = 0.87; 95% CI 0.78–0.96; *p* = 0.0001). Survival analysis, based on gene expression (*n* = 375; 25-y follow-up), confirmed that low asporin levels are associated with a reduced likelihood of survival (hazard ratio = 0.58; 95% CI 0.37–0.91; *p* = 0.017). Although these data highlight the potential of asporin to serve as a prognostic marker, confirmation of the clinical value would require a prospective study on a much larger patient cohort.

**Conclusions:**

Our data show that asporin is a stroma-derived inhibitor of TGF-β1 and a tumor suppressor in breast cancer. High asporin expression is significantly associated with less aggressive tumors, stratifying patients according to the clinical outcome. Future pre-clinical studies should consider options for increasing asporin expression in TNBC as a promising strategy for targeted therapy.

## Abstract 2

Andrei Turtoi and colleagues describe a mechanistic role for stroma-derived asporin in breast cancer development.

## Editors' Summary

**Background:**

Breast cancer is the most common cancer in women worldwide. Nearly 1.7 million new cases were diagnosed in 2012, and half a million women died from the disease. Breast cancer begins when cells in the breast that normally make milk (epithelial cells) acquire genetic changes that allow them to divide uncontrollably and to move around the body (metastasize). Uncontrolled cell division leads to the formation of a lump that can be detected by mammography (a breast X-ray) or by manual breast examination. Breast cancer is treated by surgical removal of the lump or, if the cancer has started to spread, by removal of the whole breast (mastectomy). After surgery, women often receive chemotherapy or radiotherapy to kill any remaining cancer cells, and women whose tumors express receptors for the female sex hormones estrogen and progesterone or for HER2, a growth factor receptor, are treated with drugs that block these receptors; estrogen, progesterone, and HER2 all control breast cell growth. Nowadays, the prognosis (outlook) for women living in high-income countries who develop breast cancer is generally good—nearly 90% of such women are still alive five years after diagnosis.

**Why Was This Study Done?:**

The cells surrounding cancer cells—cancer-associated fibroblasts and other components of the stroma—support cancer growth and metastasis and are good targets for new cancer therapies. But, although there is mounting evidence that cancer cells actively adapt the stroma so that it produces various factors the tumor needs to grow and spread, very few molecules produced by the stroma that might serve as targets for drug development have been identified. Here, the researchers investigate whether a molecule called asporin might represent one such target. Asporin, which is highly expressed in the stroma of breast tumors, inhibits a growth factor called TGF-β1. TGF-β1 is involved in maintaining healthy joints, but is also a key molecule in the development of metastatic breast cancer. Most particularly, it modulates an important step in metastasis called the epithelial to mesenchymal transition and it regulates “stemness” in cancer cells. Stem cells are a special type of cell that can multiply indefinitely; tumor cells often look and behave very much like stem cells.

**What Did the Researchers Do and Find?:**

Using a technique called immunohistochemistry, the researchers first showed that asporin is highly expressed in the stroma of most human breast cancers but not in normal breast tissue. Next, they showed that breast fibroblasts secrete asporin when exposed to conditioned medium from some human breast cancer cell lines (breast cancer cells adapted to grow continuously in the laboratory; conditioned medium is the solution in which cells have been grown). Specifically, conditioned medium from hormone receptor positive cells induced strong asporin expression by breast fibroblasts, whereas medium from breast cancer cells not expressing estrogen or progesterone receptors or HER2 receptors (triple-negative breast cancer cells) suppressed asporin expression. Other experiments showed that TGF-β1 secreted by breast cancer cells induces asporin expression in breast fibroblasts, and that asporin, in turn, inhibits TGF-β1-mediated induction of the epithelial to mesenchymal transition and stemness in breast cancer cells. Triple negative breast cancers appear to inhibit stromal expression of asporin at least in part via expression of the soluble signaling protein interleukin-1β. Notably, in mouse models of triple-negative breast cancer, tumors engineered to express asporin grew slower and metastasized less than tumors not expressing asporin. Finally, among women with breast cancer, asporin expression was low in triple-negative and HER2-positive tumors but significantly higher in hormone receptor positive tumors, and low asporin levels in primary breast lesions were associated with a reduced likelihood of survival independent of hormone receptor and HER2 expression.

**What Do These Findings Mean?:**

These findings suggest that asporin is a stroma-derived inhibitor of TGF-β1 and a tumor suppressor in breast cancer. Importantly, they also provide preliminary evidence that high asporin expression is associated with less aggressive tumors (hormone receptor positive tumors), whereas low asporin expression is associated with more aggressive tumors (triple negative tumors and HER2-positive tumors). Thus, asporin expression might provide a new prognostic marker for breast cancer. However, before asporin can be used as a biomarker to predict outcomes in women with breast cancer and to identify those women in need of more aggressive treatment, these findings need to be confirmed in large prospective clinical studies. If these findings are confirmed, methods for increasing asporin expression in the stromal tissues of triple negative breast cancer could be a promising strategy for targeted therapy for this group of breast cancers, which currently have a poor prognosis.

**Additional Information:**

This list of resources contains links that can be accessed when viewing the PDF on a device or via the online version of the article at http://dx.doi.org/10.1371/journal.pmed.1001871.

- The US National Cancer Institute provides comprehensive information about cancer (in English and Spanish), including detailed information for patients and professionals about breast cancer and an online booklet for patients
- Cancer Research UK, a not-for-profit organization, provides information about cancer; its detailed information about breast cancer includes sections on tests for hormone receptors and HER2, on treatments that target hormone receptors and treatments that target HER2, and on triple negative breast cancer
- Breastcancer.org is a not-for-profit organization that provides up-to-date information about breast cancer (in English and Spanish), including information on hormone receptor status, HER2 status, and triple negative breast cancer
- The UK National Health Service Choices website has information and personal stories about breast cancer; the not-for-profit organization Healthtalk.org also provides personal stories about dealing with breast cancer

## Introduction

The tumor stroma, and especially cancer-associated fibroblasts (CAFs), is emerging as a key element of cancer growth and metastasis. CAFs supply cancer cells with a plethora of growth factors, energy substrates, and immune suppressors [1–3]. In most studies to date, the CAFs and other stromal cells have been observed to support tumor growth. The reverse is naturally less evident, as tumors inhibited by the stroma do not necessarily develop. Indeed, the inability of malignant cells to properly activate the host fibroblasts and program them to serve their needs would probably result in tumor failure [4–7]. However, it is far from clear how cancer cells perform this very early reprogramming of the stroma, what the anti-tumor responses of the stromal cells to these initial events are, and why, sometimes, the battle is lost against the tumor. Our previous studies, aiming to recognize accessible tumor proteins in human renal carcinoma [8] and colon [9], pancreas [10], and breast [11] adenocarcinomas, have consistently identified an overexpression of several small leucine-rich proteoglycans (SLRPs). In the current study, we aimed to explore asporin, a member of the class I SLRP family [12], which is at present insufficiently researched in cancer.

Asporin is a secreted extracellular matrix protein that contains 380 amino acids. It was first identified in human cartilage, and its overexpression has been associated with osteoarthritis pathogenesis [13]. In normal tissues, asporin is found in articular cartilage, periodontal ligaments, the aorta, and the uterus [13,14], with no known protein isoforms reported to date. Like other SLRP family members, asporin contains a highly conserved (putative) pro-peptide sequence, has a series of leucine-rich repeats that are flanked by two cysteine residues in the C-terminal region, and has four cysteine residues that form disulfide bonds in the N-terminal domain [12]. Despite this similarity to other members of the SLRP family, in contrast to decorin and biglycan, asporin cannot be considered a typical proteoglycan because it lacks the consensus sequence necessary for glycosaminoglycan binding. Moreover, unlike other proteoglycans, asporin contains an aspartic acid repeat in its N-terminal region, polymorphisms of which have been associated with osteoarthritis [13,15]. SLRPs have been shown to be involved in several signaling pathways in which they bind to either ligands or receptors—such as bone morphogenic protein-4 (BMP-4), Wnt-I-induced secreted protein-1 (WISP-1), platelet-derived growth factor (PDGF), tumor necrosis factor-alpha (TNFα), and transforming growth factor-β1 (TGF-β1)—in the extracellular compartment [14,15].

Thus far, in tumor, high expression of asporin protein has been confirmed in pancreas [10], breast [11], prostate [16], and, recently, scirrhous gastric cancers [17]. We were intrigued by the low expression of asporin in normal human tissues, its high expression in breast carcinoma, and, particularly, its previously reported interaction with TGF-β1 in the context of osteoarthritis [15,18]. Kou et al. [18], and previously Kizawa et al. [15], demonstrated that asporin was able to bind to TGF-β1 and inhibit its ability to induce cartilage matrix gene expression. This regulation was mediated by direct binding to TGF-β1 of amino acids 159–205 of the asporin protein [18]. The reversibility of this interaction and, more importantly, its relevance in vivo to diseases such as cancer remain yet to be defined and explored using appropriate animal models. TGF-β1 is a paramount cytokine that is a potent modulator of immune evasion [19], angiogenesis [20], invasion [21], epithelial to mesenchymal transition (EMT) [22], metastasis [23], and stem cell biology [24,25]. In malignant tumors, TGF-β1 is secreted by both cancer cells and CAFs and has a demonstrated, and intriguing, dual role (pro- and anti-tumor) [26], suggesting the intervention of a more complex regulatory mechanism to modulate the spatiotemporal activity of this cytokine.

Complementing the classic “seed and soil” theory, mounting evidence shows that cancer cells actively adapt the stroma (“soil”), enabling the colonization of distant organs from the primary tumor site [1,3]. Preventing the stroma from reprogramming into a tumor-supportive environment is therefore key to a successful anti-cancer therapy. However, to date, there are only a few well-characterized stromal molecules that could serve as a basis for effective drug development [1]. The present study contributes to the field by exploring the function of a new soluble stromal protein in breast cancer growth and progression. Given its previously described TGF-β1-inhibiting function in normal chondrocytes, we hypothesized that asporin may assume an important multifaceted tumor-suppressor function in breast cancer.

## Methods

### Patient Samples

The ethical committee of the University Hospital Liège approved the use of human material in the current study. All samples were obtained from the institutional biobank of the University Hospital Liège, Belgium. According to Belgian law, patients were informed that the residual material from surgical procedures could be used for research purposes, and consent is presumed as long as the patient does not oppose (opt out). CAFs were isolated from tumors of three individual breast cancer patients (all female, mean age 55 y; tumors: estrogen receptor [ER] positive/progesterone receptor [PR] positive/HER2 negative, grade 2, Ki67+ [40%–60%]). For breast cancer, two collections of paraffin-embedded material and one set of freshly sampled tumors with adjacent non-tumoral tissue were used. The analysis of asporin expression in different subtypes of breast ductal adenocarcinoma was conducted retrospectively on a series of 180 patients (45 per subgroup). The correlation of asporin with the patient outcome was examined using an additional set of 60 patients, who had an average follow-up of 10 y. In this cohort, 30 cases had developed distant metastases (referred to as poor outcome), whereas the remainder showed no evidence of disease progression (good outcome) following the removal of the primary tumor. Other than as mentioned above, the same inclusion criteria were used for both cohorts. The inclusion criteria were as follows: (i) tumor lesion of 0.5–50 mm diameter, (ii) tumor lesion confirmed to be a breast adenocarcinoma of grade 2 and 3 after histology analysis, (iii) patient had no treatment before surgery, and (iv) patient had no metastasis at the time of surgery. Pathological characteristics for both patient groups are outlined in S1 and S2 Tables.

### Immunohistochemistry

Formalin-fixed paraffin-embedded tissue sections were prepared from primary breast cancer lesions (see “Patient Samples” above) and from xenografted tumors (see “In Vivo Study” below). Tissue samples were sliced from paraffin blocks (5-μm sections), deparaffinated three times in xylene for 5 min and hydrated in a methanol gradient (100%, 95%, 70%, and 50%). Blocking of unspecific peroxidase activity was performed for 30 min with 3% H_2_O_2_ and 90% methanol. Citrate buffer (10 mM [pH 6]) was used for antigen retrieval. The following antibodies were used: asporin (rabbit anti-ASPN, dilution 1:150, Sigma-Aldrich, catalog no. HPA008435), IL-1β (dilution 1:80, Santa Cruz Biotechnology, catalog no. sc-7884), Ki67 (Ventana Medical Systems, catalog no. 790–4286), and vimentin (Ventana Medical Systems, catalog no. 760–2512). The incubation with the primary antibody was performed overnight at 4°C. Following this, the slides were washed with PBS for 10 min. The biotinylated secondary antibody was incubated initially for 30 min and subsequently with the avidin biotin complex kit (Dako, catalog no. X0590) for an additional 30 min. 3,3′-diaminobenzidine tetrachlorhydrate dihydrate (DAB) with 5% H_2_O_2_ was used for detection. The slides were counter-stained with hematoxylin.

The quantification of protein expression was performed by two independent observers (average values are reported) and according to previously published methodology [27] with minor modifications to the scoring scale. Briefly, each immunohistochemistry (IHC) slide was assessed for the intensity of the staining of the tumor stroma using the following scale: 0 = no staining, 1 = weak, 2 = moderate, and 3 = strong. The tissue was further evaluated for the extent of positivity (percent positive stroma in the tumor) using the following scale: 1 = 0%–25%, 2 = 25%–50%, 3 = 50%–75%, and 4 = 75%–100%. The values obtained by each of the two scales were multiplied to yield a composite value called the IHC score. Pictures of representative fields were taken under a Leica DMRB light microscope. The details on statistical analysis are outlined below.

### Isolation of Primary Fibroblasts

NBFs were derived from mammary reduction specimens, whereas CAFs were collected from ductal adenocarcinoma tissue material. Fibrous areas of normal breast tissue and breast tumors were cut into small pieces and digested for 18 h at 37°C in Dulbecco’s Modified Eagle’s Medium (DMEM, Lonza) supplemented with 10% FBS, 100 U/ml streptomycin, 100 μg/ml penicillin, 2.5 μg/ml Fungizone (Gibco BRL, Life Technologies), 150 U/ml hyaluronidase (Sigma-Aldrich), and 200 U/ml collagenase type III (Gibco BRL). The digested tissue was centrifuged at 100*g* and plated in T25 tissue culture flasks with DMEM and 20% FBS.

### Cell Culture

Human epithelial breast cells MCF-7, T47D, ZR751, SKBR3, BT-474, MDA-MB-231, BT-549, and MCF-10A were obtained from ATCC. MDA-MB-468 cells were a kind gift from Dr. Sebastiano Andò (Laboratory of General Pathology, Department of Pharmacy and Health and Nutritional Sciences, University of Calabria), and EpRAS murine breast cancer cells were a kind gift of Dr. Sabine Macho-Maschler (Department of Molecular Genetics, Faculty of Veterinary Medicine, University of Vienna). MCF-7 cells were maintained in Eagle’s Minimum Essential Medium (Lonza) supplemented with 10% FBS, 1% Non-Essential Amino acid Solution (Lonza), and 2.5 mM L-glutamine (Lonza). T47D, ZR751, MDA-MB-231, and MDA-MB-468 cells were maintained in DMEM (Lonza) supplemented with 10% FBS and 2.5 mM L-glutamine (Lonza). SKBR3 cells were maintained in McCoy’s medium (Lonza) supplemented with 10% FBS. BT-474 cells were maintained in RPMI 1640 (Lonza) supplemented with 10% FBS and 1 mM sodium pyruvate, and BT-549 cells were maintained in RPMI 1640 (Lonza) supplemented with 10% FBS and 1 μg/ml bovine insulin (Sigma-Aldrich). EpRAS cells were maintained in DMEM supplemented with 10% FBS and 1 mM sodium pyruvate (Lonza). MCF-10A cells were maintained in DMEM containing 5% horse serum, 2.5 g/l glucose, 20 ng/ml EGF, 100 ng/ml cholera toxin, 0.01 ng/ml insulin, and 0.5 μg/ml hydrocortisone (all from Sigma-Aldrich). Normal breast fibroblasts (NBFs) and CAFs were maintained in DMEM supplemented with 10% FBS and 2.5 mM L-glutamine. Sub-confluent cultures (70%–90% confluence) of low passages (until passage 8) were utilized for all experiments. Conditioned medium (CM) from breast cancer cell lines was obtained following 48 h of incubation of 80% confluent cells in serum-free medium. For starvation, DMEM was used for all the cell lines. Cancer cell CM was collected, centrifuged for 5 min at 150*g* at room temperature, and then added to NBF and CAF monolayers (both cell types were pre-starved in serum-free medium for 24 h) for an additional 48 h. Following this, the NBF and CAF monolayers were washed two times with PBS and then either lysed with RIPA buffer for Western blot analysis or used for RNA extraction.

### Western Blot Analysis and ELISA

The tissues were obtained immediately after surgery from patients undergoing breast cancer resection or from primary tumors from mice. The samples were frozen in liquid nitrogen and crushed into powder. CM samples were concentrated 10-fold using Amicon Ultra centrifugal filters (Millipore, catalog no. UFC500324). Total proteins from tissues or cells were extracted using RIPA buffer (50 mM Tris-HCl [pH 7.5], 150 mM NaCl, 1% Triton X-100, 0.5% sodium deoxycholate, 0.2% sodium dodecyl sulfate, and protease/phosphatase inhibitor cocktail; Thermo Scientific, catalog no. 78440). The protein content was determined using the Pierce BCA Protein Assay Kit (Thermo Scientific, catalog no. 23225). Twenty micrograms of proteins or concentrated CM was supplemented with Laemmli buffer (0.1% 2-mercaptoethanol, 0.0005% bromophenol blue, 10% glycerol, 2% SDS in 63 mM Tris-HCl [pH 6.8]) and were separated on 10% polyacrylamide denaturing gel and transferred to nitrocellulose membranes. The following antibodies were used: anti-ASPN pAb (dilution 1:500, Sigma-Aldrich, St. Louis, MO, USA, catalog no. HPA008435), anti-SMAD2/3 mAb (dilution 1:1,000, Cell Signaling Technology, catalog no. 8685), anti-phospho-SMAD2/3 pAb (dilution 1:500, Cell Signaling Technology, catalog no. 8828), anti-E-cadherin mAb (dilution 1:1,000, BD, catalog no. 610181), anti-human vimentin mAb (dilution 1:1,000, Sigma-Aldrich, catalog no. V6389), anti-mouse vimentin mAb (dilution 1:1,000, Cell Signaling Technology, catalog no. 5741), and anti-HSC70 mAb (dilution 1:30,000, Santa Cruz Biotechnology, catalog no. sc-7298).

For ELISA assay, serum-free CM from cell lines was collected after 48 h of incubation, clarified by centrifugation, and then activated and processed using the TGF-β1 ELISA kit (R&D Systems, catalog no. DB100B) following the manufacturer’s instructions. Data were normalized according to the number of cells.

### Gene Expression Analysis

Total RNA was isolated with the High Pure RNA Isolation Kit (Roche, catalog no. 11828665001). One microgram of total RNA was reverse-transcribed using the Transcriptor First Strand cDNA Synthesis Kit (Roche, catalog no.04897030001) according to the manufacturer’s instructions. The cDNAs (100 ng) were mixed with primers (0.5 μM), human UPL-probe system (0.2 μM) (Roche, catalog no. 04683633001), and 2× FastStart Universal Probe Master mix (Roche, catalog no. 04914058001) and analyzed in triplicate. Quantitative real-time PCR (qRT-PCR) was performed using the LightCycler 480 system (Roche) and the corresponding manufacturer software. The following cycling conditions were used: 95°C for 10 min then 40 cycles of 95°C (15 s) and 60°C (1 min). Sequences of asporin primers were as follows: forward 5′-GGTGGATAACTTCTACTTTTAGGAGGA-3′ and reverse 5′-AAGAAGGGTTT-GGCAGAGC-3′ and UPL probe #72. The relative gene expression levels were normalized using 18S rRNA content (Life Technologies, catalog no. 4310893E).

### Treatment with rTGF-β1, rASPN, ASPN Peptide Fragment, IL-1β, and IL-1RA

Cells were starved in serum-free medium for 16 h and then treated for 15 min with a recombinant active form of TGF-β1 (Roche, catalog no. 11412272001) and/or recombinant human asporin (a kind gift of Targetome) and a synthetic peptide fragment of asporin protein (amino acids 159–205, H-NQLSEIPLNLPKSLAELRIHENKVKKIQKDTFKGMNALHVLEMSAN-OH) (Bachem). TGF-β1, recombinant asporin, and asporin peptide were dissolved in PBS and used at different concentrations according to the experimental setup (for respective concentrations and treatment times see figure captions). When used together, asporin peptide and recombinant TGF-β1 were pre-incubated for 1 h at 37°C before being added to the cells.

Analysis of the EMT was conducted using Ras-transformed mammary epithelial cells (EpRAS). The EMT induction was performed as previously described [28]. Briefly, 5 × 10^4^ cells/well were plated in a six-well plate and were grown in the presence or absence of TGF-β1 and/or asporin peptide. Treatments were repeated every day, following medium change, and the cells were cultured for 10 d. During this period the cells were re-plated every 3 d at 5 × 10^4^ cells/well.

For IL-1β experiments, starved NBFs and CAFs were incubated with CM or serum-free medium supplemented with 5 ng/ml TGF-β1 in the presence or absence of 0.1–0.5 ng/ml IL-1β (Peprotech, catalog no. 200-01B) for 48 h. IL-1β activity was blocked by pretreating starved NBFs with 40 ng/ml IL-1RA (Peprotech, catalog no. 200-01RA) for 1 h, followed by the addition of MDA-MB-231 CM to the pretreated NBFs for 48 h.

### Migration Assay

EpRAS mouse breast cancer cells were pretreated for 10 d with TGF-β1 and/or asporin peptide (P159–205), as described above. At the end of this period, 1 × 10^5^ cells were suspended in serum-free medium (0.1% BSA, 1% penicillin/streptomycin) and seeded into the upper part of a Transwell filter (diameter 6.5 mm, pore size 8 μm; Costar, catalog no. 3422). The lower compartment was filled with DMEM containing 1% pen/strep and 10% FBS. Following 16 h of incubation at 37°C, migrating cells were fixed and stained with Diff-Quick kit (Reagena, catalog no. 102164). Pictures of each insert were taken at 5× magnification, and migrating cells were counted using ImageJ software (US National Institutes of Health).

### Quantification of Stem Cells

#### EpRAS cells

On the tenth day of treatment with TGF-β1 and/or asporin peptide, the cells were harvested with trypsin, and 2.5 × 10^5^ cells were suspended in 25 μl of PBS and labeled with 1/100 (0.01 mg/ml) anti-CD24 (biotinylated; BioLegend, catalog no. 101803) for 1 h at 4°C. Following a wash, the cells were further labeled with 1/100 (0.01 mg/ml) anti-CD44 (PE-labeled; eBioscience, catalog no. 12–0441) and 1/1,000 streptavidin-FITC (Invitrogen, catalog no. SA100-02) for 1 h at 4°C. Following this, the cells were washed two times, and 1/50 7-aminoactinomycin D (7-AAD; BD-Pharmingen, catalog no. 51-68981E) was added for 10 min. The cell suspensions were analyzed using a FACSAria flow cytometer (BD Biosciences). Stem cells were quantified by evaluating the percentage of 7-AAD^neg^, CD44^high^/CD24^low^ cell population.

#### MDA-MB-468 xenografts

Tumors were removed from NOD-SCID mice 7 wk post-implantation, minced, and digested in a solution of hyaluronidase (300 μg/ml) (Sigma-Aldrich, catalog no. H-3506) and collagenase I (1.75 mg/ml) (Sigma-Aldrich, catalog no. C0130) in HSSB (Life Technologies, catalog no. 14025–050) and incubated for 2 h at 37°C. 5 × 10^5^ isolated cells were assayed for ALDH activity using the Aldefluor kit (Stemcell Technologies, catalog no. 01700), according to the supplied protocol. Human CD44 R-PE conjugate (Life Technologies, catalog no. MHCD4404) and human CD24 PE-Alexa Fluor 610 conjugate (Life Technologies, catalog no. MHCD2422) were incubated (both at 1/100 dilution) with the cell suspension for 1 h at 4°C. Following this, the cells were washed two times and 1/50 7-AAD was added for a further 10 min. The cell suspension was analyzed using a FACSAria flow cytometer (BD Bioscences). Stem cells were quantified by evaluating the percentage of cell populations characterized by two separate signatures: (i) 7-AAD^neg^, ALDH^+^ and (ii) 7-AAD^neg^, CD44^high^/CD24^low^.

### Stable Clones

The MDA-MB-468 cells and NBFs were modified to express luciferase and asporin (MDA-MB-468-*aspn*; NBF-*aspn*) or green fluorescent protein (MDA-MB-468-*ctrl*; NBF-*ctrl*). Briefly, pLenti6-IRES-Luciferase plasmid was generated by cloning IRES (Internal Ribosome Entry Site) and firefly (*Photinus pyralis*) luciferase sequences into a lentiviral plasmid using the pLenti6/V5 Directional TOPO Cloning Kit (Invitrogen) in order to allow the expression of the luciferase gene under the control of CMV promoter. The 1,152 bp of the *Homo sapiens* asporin (ASPN, transcript variant 1) cDNA (NM_017680.4) was synthetized by GenScript and then cloned into pLenti6-IRES-Luciferase to obtain the pLenti6-ASPN-IRES-Luciferase plasmid for dual ASPN and luciferase expression. Lenti-X 293T cells (Clontech, 632180) were co-transfected with pSPAX2 (Addgene, plasmid #12260), a VSV-G-encoding vector, along with pLenti6-IRES-Luciferase or pLenti6-ASPN-IRES-Luciferase plasmids. 48 h and 72 h post-transfection, viral supernatants were collected, filtrated, and concentrated 100× by ultracentrifugation. The lentiviral vectors were then titrated with qPCR Lentivirus Titration Kit (ABM, LV900). Finally, the MDA-MB-468 cells and NBFs were transduced with 30 viral vectors per cell. After 48 h, positively transduced cells were selected with 10 μg/ml blasticidin (Invivogen, ant-bl-1). The cell culture supernatants were checked for the absence of replication-competent lentivirus before employing cells in vivo.

### In Vivo Study

All experimental procedures used in the current work were performed in accordance with the ARRIVE ethical guidelines [29] and were reviewed and approved by the Institutional Animal Care and Ethics Committee of the University of Liège (Belgium). The experimentation adhered to the *Guide for the Care and Use of Laboratory Animals* prepared by the Institute of Laboratory Animal Resources of the National Research Council and published by National Academies Press, as well as to European and local legislation. NOD-SCID mice were purchased from Janvier Labs and housed in the animal facility of the University of Liège under standard conditions (12 h light/dark cycle, lights on at 7 a.m.). They were acclimated to the room 1 wk before the beginning of the experiment. Food and water were provided ad libitum. Tumor development was monitored at weekly intervals using in vivo imaging and caliper volume measurement (primary experimental outcome). For in vivo imaging, an intra-peritoneal injection of luciferin (Promega, catalog no. E1605) was given to the mice, and the signal was accrued using a Xenogen IVIS 200 imaging system (Caliper Life Sciences). Tumor volumes were calculated by acquiring the length (*L*), width (*W*), and height (*H*) of the xenografts and employing the formula *V* = (*L*/2) × (*W*/2) × (*H*/2) × π × (4/3). For the follow-up (size-matched experiments), the tumors were surgically removed at the target volume of 200–250 mm^3^ (reached in 6 to 12 wk, depending on the experimental condition). For the surgical removal of the primary tumor, mice were anaesthetized using 75 mg/kg of ketamine (Ceva) and 10 mg/kg of xylazine (Rompun, Bayer). Lung metastases were quantified in necropsy material using either IHC (vimentin staining) or Alu-PCR (see below), depending on the required sensitivity (secondary experimental outcome). Quantification of IHC was performed in serial paraffin sections by evaluating two parameters: (i) the frequency of metastatic foci (each individual cell or group of cells was counted as one deposit) and (ii) the size of the metastatic deposits (grouping them into three categories: <10 cells, 10–20 cells, and >20 cells). Statistical analysis was performed as described below.

#### Fibroblast/MDA-MB-468 co-injection xenografts

5 × 10^5^ luciferase-positive NBF-*aspn* or-*ctrl* cells were mixed with 5 × 10^5^ luciferase-positive MDA-MB-468 cells, suspended in cell culture medium, mixed (1:1) with growth-factor-reduced Matrigel (BDBiosciences, catalog no. 356230), and inoculated subcutaneously into the flanks of 5-wk-old NOD-SCID mice. Mice were randomly allocated to one of the two groups. Following tumor removal the mice were monitored for metastasis development for an additional 2 wk. Twenty mice were used in total.

#### MDA-MB-468 xenografts

2 × 10^6^ million luciferase-positive MDA-MB-468-*aspn* or-*ctrl* cells were suspended in cell culture medium, mixed (1:1) with growth-factor-reduced Matrigel (BD Biosciences), and inoculated subcutaneously into the flanks of 5-wk-old NOD-SCID mice. Mice were randomly allocated to one of the two groups. After the resection of the primary tumor, the follow-up for metastasis development was conducted for an additional 3 wk. Eighty mice were used in total.

### qRT-PCR for Human Alu Sequences

Genomic DNA was extracted from 10 mg of lung tissue using the High Pure PCR Template Preparation Kit (Roche, catalog no. 11796828001), according to the manufacturer’s instructions. A standard curve was generated by serially diluting MDA-MB-468 cells in 10 mg of normal mouse lung tissue, followed by DNA extraction. Next, 20 ng of DNA was mixed with primers against human Alu sequences (forward 5′-CATGGTGAAACCCCGTCTCTA-3′ and reverse 5′-GCCTCAGCCTCCCGAGTAG-3′) or with primers for human/mouse GAPDH, which was used as a normalizator (forward 5′-CAGCGACACCC-ACTCCTCCACCTT-3′ and reverse 5′-CATGAGGTCCACCACCCTGTTGCT-3′) [30]. All primers were used at 0.5 μM final concentration. Finally, to the DNA/primer mix, 2× FastStart Universal SYBR Green Master mix (Roche, catalog no. 04913850001) was added. The Alu sequences were amplified using the LightCycler 480 system (Roche) and the following cycling conditions: 95°C for 10 min followed by 40 cycles of 95°C (15 s) and 60°C (1 min).

### Survival Analysis and Gene Expression Patterns

Kaplan-Meier survival curves were plotted with publicly deposited gene expression data (EGA and TCGA) originating from 375 untreated breast cancer patients and using Kaplan-Meier Plotter, which integrates statistical analysis (http://kmplot.com/analysis) [31]. All settings were left at default values except the following ones: gene symbol (ASPN; 219087_at), survival (OS), auto select best cutoff (on), and include systemically untreated patients (on).

Expression of asporin at the mRNA level was quantified in 1,280 tumor samples from six different molecular subtypes using GOBO and publicly deposited gene expression datasets (http://co.bmc.lu.se/gobo/gsa.pl) [32]. Asporin mRNA expression was also quantified in tumors of different grades (*n* = 1,411) using the same tool. Finally, GOBO was also used to estimate IL-1β expression levels in different breast cancer cell lines (http://co.bmc.lu.se/gobo/gsa_cellines.pl). All statistics concerning Kaplan-Meier Plotter and GOBO data analysis were reported as calculated by the respective software and are detailed elsewhere [31,32].

### Statistical Analysis

Unless otherwise indicated, statistical analysis was performed using a two-sided, unpaired Student’s *t*-test, assuming equal variances using GraphPad Prism (version 5.01, GraphPad Software). The *t*-test was used because data followed a normal distribution (Shapiro-Wilk test, threshold 0.05). For IHC evaluation, box plots were generated using SigmaPlot (version 11.0, Systat). Testing of statistical significance was performed using a Mann-Whitney U test because the data did not follow the normal distribution (Shapiro-Wilk test, threshold 0.05). The receiver operating characteristic (ROC) curve was generated using GraphPad Prism.

## Results

### Asporin Has Low or No Expression in Most Normal Tissues and Is Overexpressed in Breast Cancer

**Fig 1:**
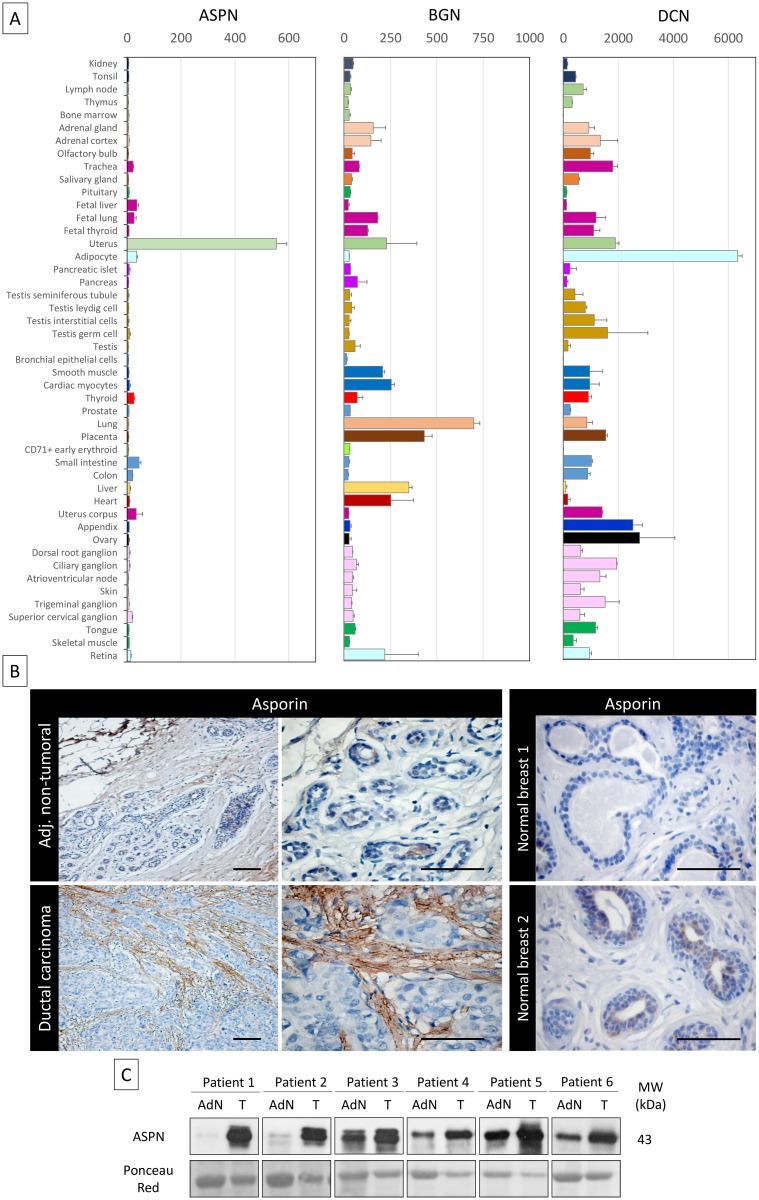
Asporin is overexpressed in breast cancer tissues. (A) Tissue-specific pattern of mRNA expression of asporin (ASPN), biglycan (BGN), and decorin (DCN). Source: BioGPS (http://biogps.org). The data are presented as mean ± standard deviation (SD). (B) Representative IHC staining of asporin expression in ductal carcinoma and adjacent non-tumoral breast tissue (left panel) and normal breast tissue obtained from patients undergoing mammary reduction surgery (right panel). Asporin is almost exclusively expressed in breast cancer lesions, while a very low signal is detectable in the adjacent non-tumoral regions. Normal breast tissues are negative. Images of representative fields were taken at 100× and 400× magnification. (C) Western blot analysis of asporin expression in tumoral breast cancer tissues (T) and the adjacent normal counterpart (AdN) of six ductal adenocarcinoma patients. Ponceau red staining was used as loading control.

We analyzed a publicly available gene expression repository (BioGPS, Scripps Research Institute) and compared the gene expression profiles in normal tissues of asporin and two other well-studied members of the SLRP family, biglycan and decorin (Fig 1A). The analysis showed that both biglycan and decorin are expressed in many normal tissues, whereas asporin expression was very low or not detected in most normal tissues, except the uterus. Next, we analyzed asporin expression using IHC in breast ductal adenocarcinoma (*n* = 30) as well as in adjacent non-tumoral tissue and normal breast tissue from breast reduction surgery (*n* = 10) (Fig 1B). Strong asporin expression was detectable in the stroma of the cancer lesions, with epithelial cancer cells being negative for asporin expression. Adjacent non-tumoral tissue showed a moderate positivity in the extracellular matrix and no positivity in non-tumoral epithelial cells. Healthy breast tissue was negative for asporin expression. Western blot analysis on fresh tissue extracts from matched tumoral and adjacent non-tumoral parts of the resected breast specimens confirmed our IHC observations (Fig 1C).

### Breast Fibroblasts Secrete Asporin after Their Activation by Cancer Cells

**Fig 2:**
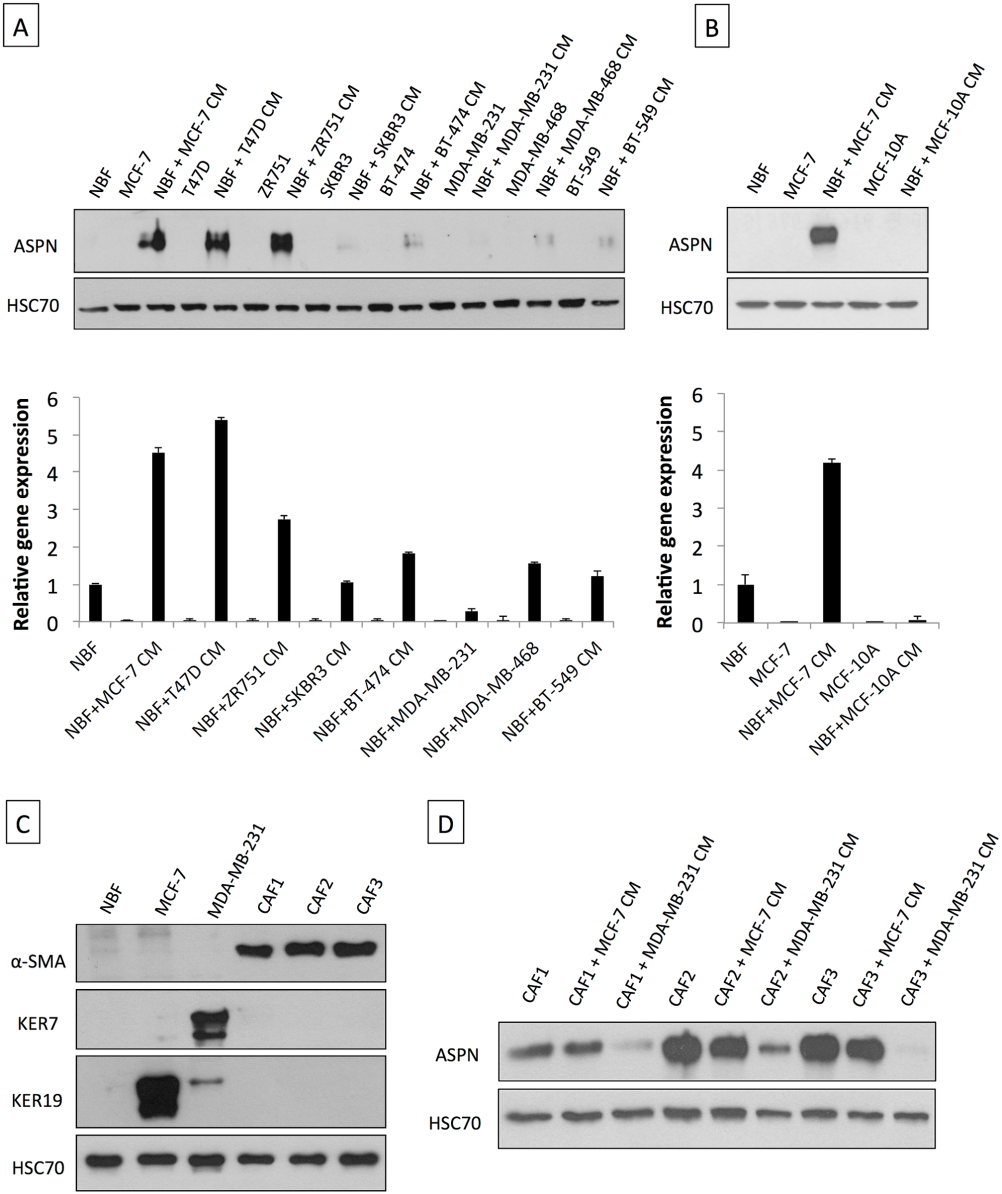
Asporin is produced by breast fibroblasts in response to conditioned medium from breast cancer cells. (A) Western blot of total cell extracts (upper panel) and qRT-PCR analysis for asporin expression (lower panel) in breast cancer cell lines and NBFs incubated for 48 h with CM collected from a panel of breast cancer cells. (B) Western blot of total cell extracts (upper panel) and qRT-PCR analysis of asporin expression (lower panel) in non-cancerous epithelial breast cell line MCF-10A cells and NBFs incubated for 48 h with CM collected from MCF-10A. Fibroblasts treated with MCF-7 CM were used as the positive control for asporin expression induction. (C) Validation of NBFs and CAFs isolated from patient material. MCF-7 and MDA-MB-231 cells were used as epithelial controls. (D) Western blot analysis of asporin expression in total cell extracts of CAFs obtained from three different patients and treated with the CM of breast cancer cell lines. (A and B): The data are presented as mean ± SD. All panels: HSC70 was used as loading control; Western blots show representative data of three independent experiments.

Owing to the observations made above, in which asporin was found deposited in the extracellular matrix of the tumor, we sought to investigate which cells are responsible for producing the protein. As demonstrated in Fig 2A, none of the human breast cancer cell lines tested showed detectable asporin expression levels (both protein and mRNA). NBFs isolated from the mammary tissue of healthy individuals responded to the CM of several breast cancer cell lines by expressing asporin. The results indicated that tumorigenic and highly metastatic triple-negative breast cancer (TNBC) cells of the basal-like subtype (e.g., MDA-MB-231 and MDA-MB-468) [33–35] did not induce asporin expression in NBFs. This was different in noninvasive luminal-like hormone receptor (HR) positive cell lines (e.g., T47D and MCF-7) [33–35], which activated very high asporin expression in NBFs. Both observations were confirmed at the protein and gene expression levels. Similar experiments with immortalized, non-transformed mammary epithelial cells (MCF-10A) demonstrated that such cells are unable to induce asporin expression in NBFs (Fig 2B). We next sought to examine whether CAFs would express asporin following their isolation from cancer tissue and whether they would react similarly to NBFs when exposed to the CM of breast cancer cells. We isolated CAFs from three breast cancer patients and validated these as pure CAF populations, negative for cytokeratins and overexpressing α-smooth muscle actin (Fig 2C). As shown in Fig 2D, CAFs isolated from HR+ tumors expressed high levels of asporin, which they were able to maintain in vitro (for several weeks) without the need to be in contact with cancer cells. However, CAFs challenged with the CM of TNBC cells (MDA-MB-231) responded by lowering asporin expression. The CM of HR+ cells (MCF-7) was unable to further increase asporin levels in CAFs.

### TGF-β1 Induces Asporin Expression in Fibroblasts while IL-1β Suppresses It

**Fig 3:**
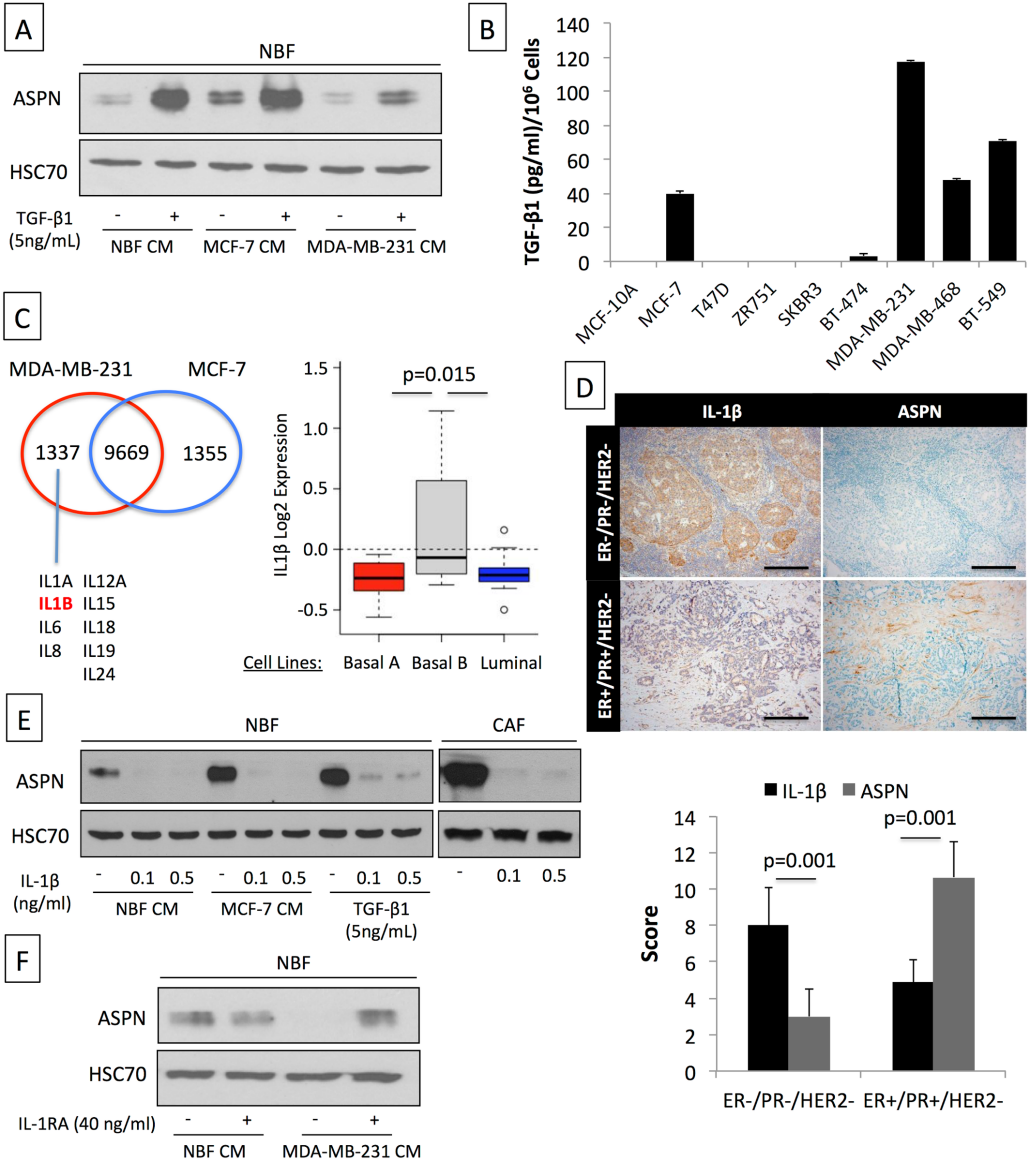
Asporin expression is induced by TGF-β1 and suppressed by IL-1β. (A) Western blot analysis of asporin expression in NBFs treated for 24 h with TGF-β1 with or without CM of MCF-7 and MDA-MB-231 breast cancer cells. (B) ELISA quantification of TGF-β1 levels secreted by human breast cancer cell lines during 48 h. (C) Differential in silico analysis of MCF-7 and MDA-MB-231 gene expression identifies a cluster of interleukins that are uniquely expressed in MDA-MB-231 cells (left panel). Analysis of IL-1β mRNA expression using GOBO (http://co.bmc.lu.se/gobo/gsa.pl) across a panel of breast cancer cell lines [35] subdivided into three subtypes (right panel). (D) Representative IHC analysis (upper panel) of IL-1β and asporin expression in a cohort of breast cancer patients, subdivided into two main subtypes (*n* = 20). Scoring and statistics (lower panel) were performed as outlined in the Methods section. (E) Inhibition of basal, MCF-7 CM-induced, and TGF-β1-induced asporin expression by IL-1β treatment of NBFs and CAFs. (F) Reversion of MDA-MB-231 CM inhibitory effect on asporin expression using IL-1β natural inhibitor IL-1RA. (B and D): The data are presented as mean ± SD. All panels: Western blots show representative data of three independent experiments.

In order to further understand which soluble factors could modulate asporin expression in fibroblasts, we first tested the cytokine TGF-β1. As shown in Fig 3A, TGF-β1 induced asporin expression in NBFs, both under basal conditions and in the presence of CM from MCF-7 cells. In sharp contrast to this, the presence of CM from MDA-MB-231 cells strongly inhibited the ability of TGF-β1 to induce asporin in NBFs. Despite this, our measurements showed that MDA-MB-231 cells, including other TNBC cells, are the highest producers of TGF-β1 among different breast cancer cells (Fig 3B). These findings raised the question of the specific mechanism by which TNBC cells inhibit asporin expression while still producing large quantities of TGF-β1. To further clarify this, we used publicly deposited gene expression data (GEO datasets GSE56265 [two replicates for each cell line] and GSE41445 [three replicates]) comparing the mRNA expression of MDA-MB-231 and MCF-7 cells [36,37]. The summary of the results is shown in Fig 3C. We identified 1,337 genes that were uniquely expressed in MDA-MB-231 cells. Considering that the CM of TNBC cells is able to convey the suppression of asporin expression without the need for cell-to-cell contact, we hypothesized that the effect must be mediated through a soluble protein. We therefore focused only on the genes whose products are known to be soluble proteins. The analysis highlighted a strong cluster of interleukins that were expressed in MDA-MB-231 cells. We next sought to verify which of the observed interleukin genes would discriminate between HR+ (luminal) and TNBC (basal) cells. This analysis was performed using GOBO [32], which compares the profiles of 51 breast cancer cell lines representing different molecular subtypes [35]. The results indicated that IL-1β could be of interest because it is highly expressed in cell lines of the basal-b subtype, which includes MDA-MB-231 cells (Fig 3C, right panel). Encouraged by these in silico findings, we sought to verify the expression of IL-1β in patient material from different breast tumor subtypes and compare this with asporin expression. IHC analysis of ductal adenocarcinoma cases (*n* = 20) demonstrated that IL-1β expression was significantly increased in TNBC compared to HR+ tumors (Fig 3D). In contrast to this, asporin expression in serial sections of the same tissues followed an inverse trend, with the highest expression in HR+ and the lowest in TNBC tumors. Next we tested whether recombinant human IL-1β could suppress basal, MCF-7-induced, or TGF-β1-induced asporin expression in NBFs. Supplementing CM with IL-1β readily blocked asporin expression in NBFs and CAFs (Fig 3E). We also examined whether IL-1RA, a naturally produced IL-1β inhibitor, would overcome the inhibition of asporin expression in NBFs treated with the CM of MDA-MB-231 cells. Indeed, the pre-incubation of NBFs with IL-1RA blocked the suppressive effect of CM from MDA-MB-231 cells on asporin expression in NBFs (Fig 3F).

### Asporin Inhibits TGF-β1-Mediated SMAD2 Activation and Induction of the Epithelial to Mesenchymal Transition

**Fig 4:**
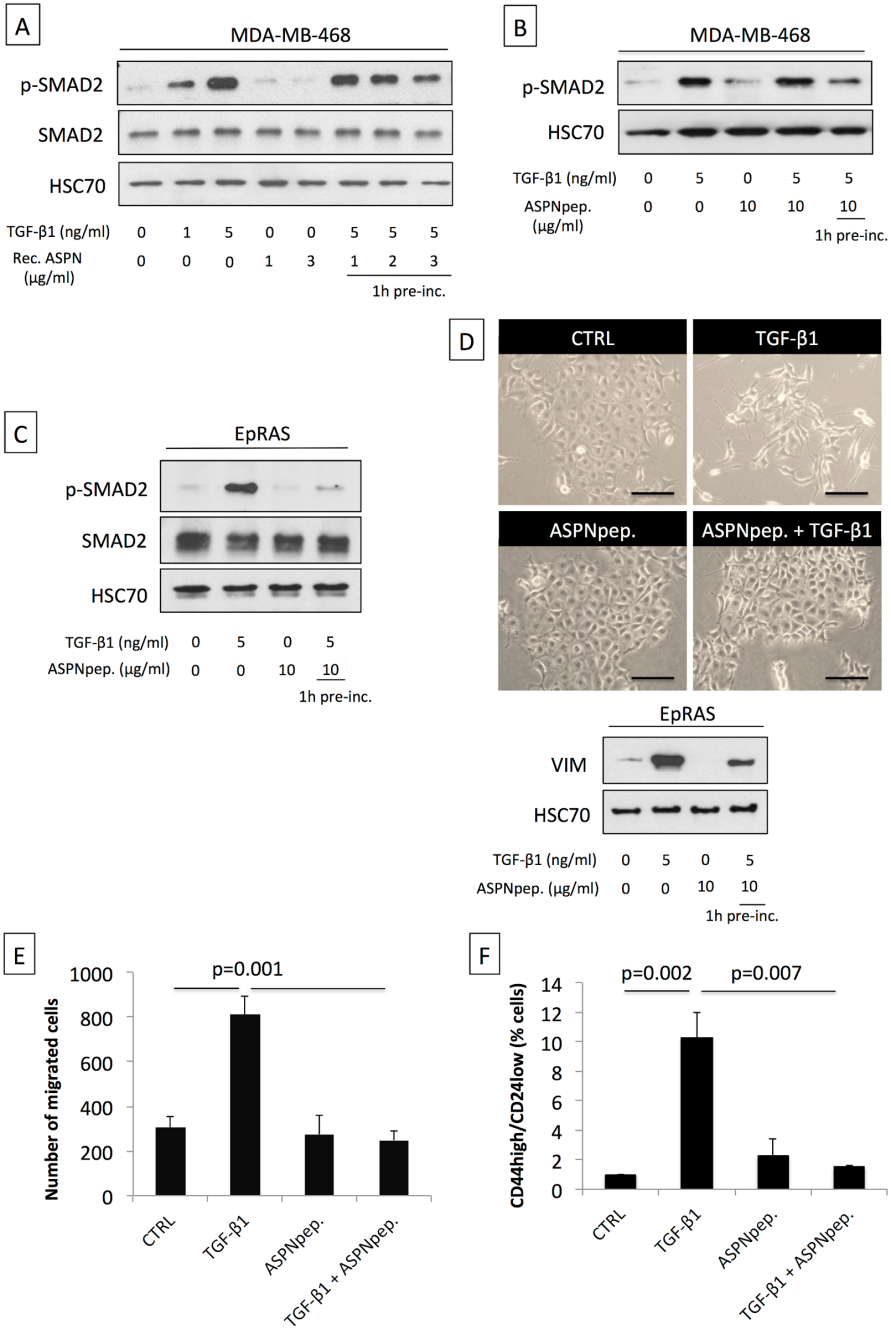
Asporin binds to TGF-β1 and inhibits its downstream signaling and function. (A) Western blot analysis of phospho-SMAD2 (p-SMAD2) and SMAD2 total protein extracts from MDA-MB-468 breast cancer cells treated for 15 min with TGF-β1 and/or human recombinant asporin (Rec. ASPN). (B) Western blot analysis of p-SMAD2 in total protein extracts from MDA-MB-468 breast cancer cells treated with TGF-β1 and/or asporin peptide corresponding to the 159–205 amino acid region (ASPNpep.). (C) Western blot analysis of p-SMAD2 and SMAD2 in total protein extracts from EpRAS cells treated for 15 min with TGF-β1 (5 ng/ml) and/or asporin peptide. (D) EMT induction in EpRAS cells in the presence of TGF-β1 and/or asporin peptide. EMT was monitored both at the phenotype level (upper panel) and using Western blot evaluation of VIM expression in total protein extracts from EpRAS cells (lower panel). (A–D): HSC70 was used as loading control. (E) Transwell migration assay of EpRAS cells pretreated with TGF-β1 (5 ng/ml) and/or asporin peptide (10 μg/ml). (F) Quantification of the CSC population in EpRAS cells following TGF-β1 and/or asporin peptide treatment. (E and F): The data are presented as mean ± SD. All panels: statistical significance was calculated using the Student’s *t*-test (as described in the Methods section). Western blots show representative data of three independent experiments.

Previous studies in chondrocytes [18] identified that the peptide region of asporin (residues 159 to 205) is relevant for the interaction with TGF-β1. We therefore tested whether recombinant asporin and the synthetically produced peptide fragment were able to inhibit TGF-β1-mediated activation of SMAD2 in breast cancer cells (Fig 4A and 4B). SMAD2 was efficiently phosphorylated upon treatment of MDA-MB-468 cells with TGF-β1. The TGF-β1 activity was inhibited when the cytokine was pre-incubated for 1 h at 37°C with increasing doses of recombinant asporin (Fig 4A). Cells treated with recombinant asporin alone showed no modulation of SMAD2 phosphorylation. Analogously to the effects observed with the recombinant protein, the peptide fragment of asporin showed an inhibitory effect on TGF-β1-induced SMAD2 phosphorylation (Fig 4B). Further data showed that parallel treatment of cancer cells with TGF-β1 and asporin peptide, without prior pre-incubation, failed to inhibit SMAD2 phosphorylation. These results are in agreement with previous findings in normal chondrocytes showing that asporin directly binds to TGF-β1, rather than acting as a competitive inhibitor for TGF-β1 receptor [28,38]. To functionally test the ability of asporin to interfere with TGF-β1-induced processes, we employed EpRAS, a murine mammary cancer cell line. EpRAS cells have an established responsiveness to TGF-β1, especially with respect to EMT and migration [28,38]. Similarly to the effects observed in MDA-MB-468 cells, asporin peptide was able to block TGF-β1-mediated phosphorylation of SMAD2 in EpRAS cells (Fig 4C). EpRAS cells are known for undergoing EMT upon stimulation with TGF-β1. Apart from the phenotypic appearance, the EMT switch can be readily observed through the up-regulation of vimentin (VIM) (Fig 4D). TGF-β1-mediated induction of VIM was weaker when TGF-β1 was pre-incubated with asporin peptide (Fig 4D). We further sought to investigate the ability of asporin to interfere with TGF-β1-induced migration of EpRAS cells (Fig 4E). Treatment of EpRAS cells with TGF-β1 induced significant cell migration, while pre-incubation of TGF-β1 with the asporin peptide significantly curbed this effect. The EMT switch has been described as relevant for the acquisition of the cancer stem cell (CSC) phenotype [24]. Therefore, we tested whether TGF-β1-induced EMT would increase the CSC population in EpRAS cells and whether this effect could be inhibited by asporin peptide (Fig 4F). Indeed, TGF-β1 treatment induced an increase of CSCs in EpRAS cells, as evidenced by the established CD44^high^/CD24^low^ breast cancer stemness signature [39]. The TGF-β1-induced increase of the CSC population was significantly inhibited when asporin peptide was pre-incubated with TGF-β1.

### Induction of Asporin Expression in Triple-Negative Breast Tumors Reduces Growth and Metastasis In Vivo

**Fig 5:**
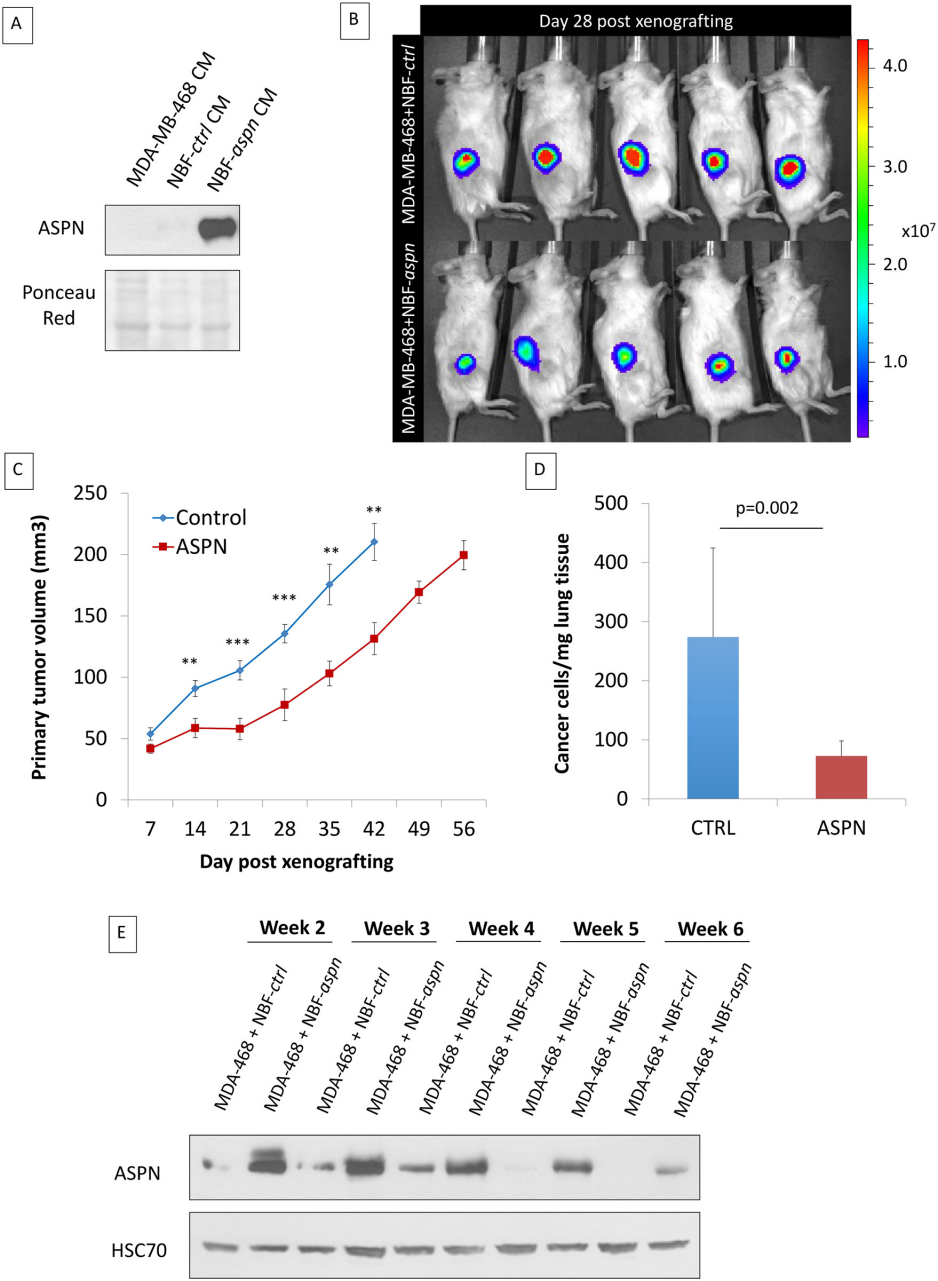
Co-injection of cancer cells and fibroblasts overexpressing asporin reduces primary breast cancer tumor growth and lung metastasis formation in vivo. (A) Western blot analysis of asporin expression in CM of MDA-MB-468 cells and in NBF stable clones used for subcutaneous injection in mice. Ponceau red is shown as loading control. (B) Bioluminescence imaging of control and asporin-expressing xenografts at day 28 after tumor engraftment. The color scale indicates the fluorescent intensity. (C) The volume (in cubic millimeters) of primary tumors measured weekly (from day 7 onwards). The data are presented as mean ± standard error of the mean (SEM) (*n* = 10 for each group). Statistical significance was calculated using Student’s *t*-test (**0.01 < *p* < 0.001; ***0.001 < *p* < 0.0001). (D) Human-specific Alu-PCR performed on genomic DNA isolated from dissected lungs was used to detect human cancer cells. The data are presented as mean ± SD. (E) Western blot analysis of asporin expression in mice primary tumors monitored for several weeks. HSC70 was used as loading control.

In order to evaluate the impact of asporin expression on TNBC growth and progression, we co-injected MDA-MB-468 cells and NBFs stably overexpressing asporin (NBF-*aspn*) subcutaneously in NOD-SCID mice (Fig 5A). The control group consisted of mice xenografted with MDA-MB-468 cells with NBFs overexpressing GFP (NBF-*ctrl*). The growth of the tumors was followed weekly, and the results indicated that the control tumors reached volumes of ~200 mm^3^ in 6 wk, whereas the asporin-overexpressing tumors grew significantly slower (day 42 post-engraftment: 78.9 mm^3^ smaller than control; 95% CI 24.3–123.4; *p* = 0.007) and required 8 wk to reach the same volume (Fig 5B and 5C). Following the resection of the primary tumor, the ability of cancer cells to colonize the lungs was assessed in a follow-up experiment. Two weeks after the removal of the primary tumors, mice were sacrificed and the lungs were collected. Assessment of metastasis formation in the lungs was performed using the Alu-PCR technique. The results evidenced a 3-fold, significant (*p* = 0.002) increase in the number of cancer cells present in the lung tissue of the control mice compared to the mice with asporin-overexpressing NBFs (Fig 5D). Previously published studies using xenografts based on co-injection of fibroblasts and epithelial cancer cells showed that human fibroblasts are rapidly displaced by murine fibroblasts in vivo [40–42]. We thus sought to verify whether asporin expression remained constant in the tumor during the present experiments. Western blot analysis of tumor tissue extracts showed that asporin expression decreased starting from week 4, reaching low levels at week 6 (Fig 5E). This result suggested the ongoing replacement of human xenografted fibroblasts by murine counterparts. This diminishing asporin expression may underestimate asporin’s effect on tumor growth and metastasis in vivo.

**Fig 6:**
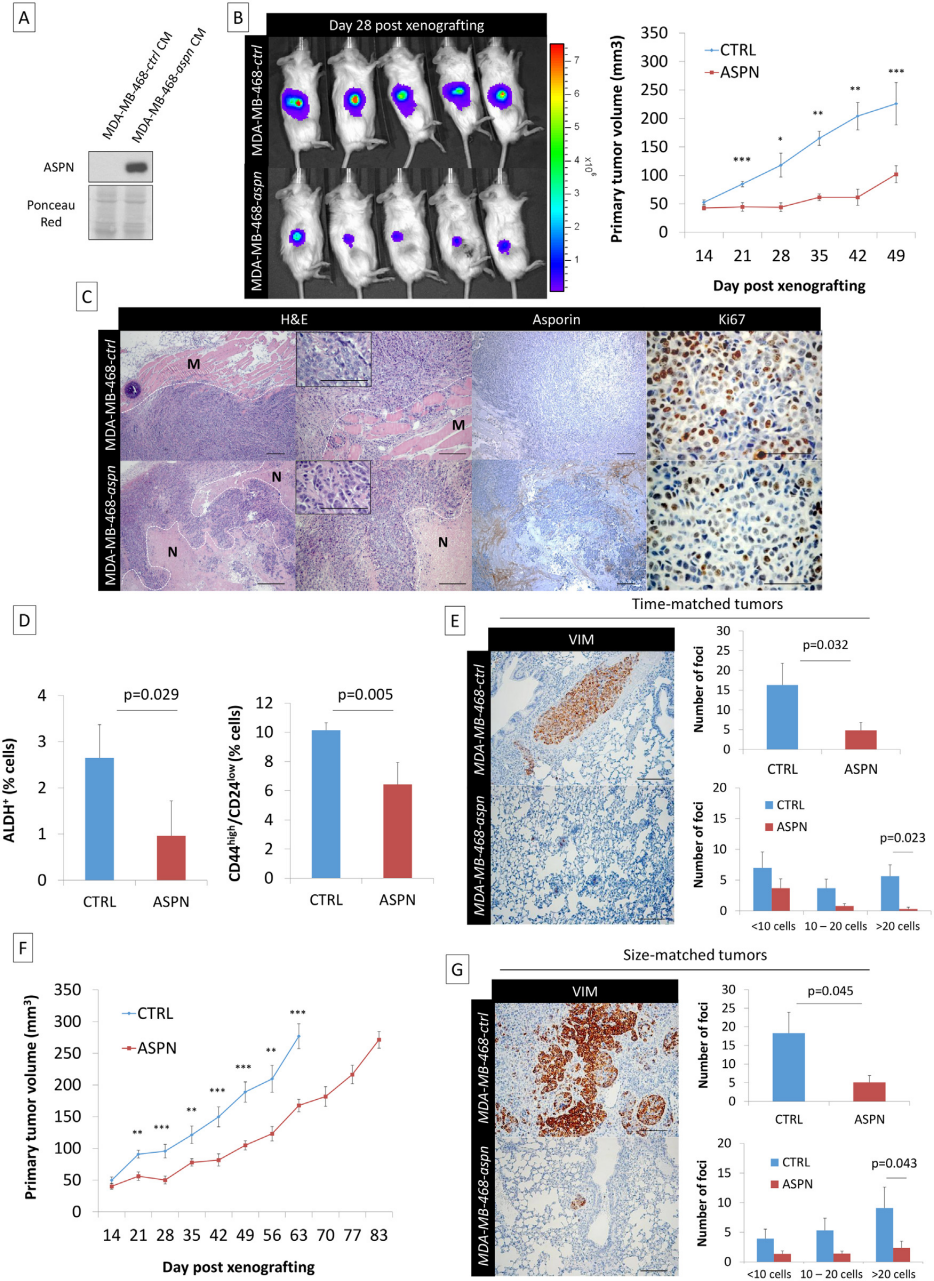
Asporin reduces primary breast cancer tumor growth and lung metastasis formation in vivo. (A) Western blot analysis of asporin expression in the CM of MDA-MB-468 stable clones expressing asporin, used for subcutaneous injection in mice. Ponceau red is shown as loading control. (B) Bioluminescence imaging of control and asporin-expressing xenografts at day 28 after tumor engraftment (left panel). The color scale indicates the fluorescent intensity. The mean (± SEM) volume (in cubic millimeters) of primary tumors measured weekly (from day 14 onwards) for the time-matched cohort is also shown (*n* = 20 for each group) (right panel). Statistical significance was calculated using Student’s *t*-test (**0.01 < *p* < 0.001; ***0.001 < *p* < 0.0001). (C) Representative hematoxylin and eosin (H&E), asporin, and Ki67 IHC staining in MDA-MB-468 xenografts collected 7 wk post-engraftment. Control xenografts consistently displayed an invasion in the muscle layer (M). An extended necrotic (N) area was present in the peri-tumoral zone of MDA-MB-468-*aspn* mice tumors. (D) Quantification of the stem cell population in xenografted tumors expressing asporin using ALDH^+^ and CD44^high^/CD24^low^ stemness markers (7 wk post-engraftment). (E) Post-operative follow-up of mice that had primary tumors removed at the same time (time-matched). (F) Mean (± SEM) volume (in cubic millimeters) of primary tumors measured weekly for the size-matched cohort (*n* = 20 for each group). (G) Post-operative follow-up of mice that had primary tumors removed at the same volume (size-matched). (E and G): IHC evaluation of vimentin in lung necropsies and quantification of metastatic deposits. All images of representative fields were taken at 40×, 100×, and 400× magnification. (D, E, and G): The data are presented as mean ± SD. Statistical significance was calculated using Student’s *t*-test.

Therefore, we next sought to engraft asporin-overexpressing cancer cells that would maintain constant asporin expression in the tumor. This was performed with stably transduced asporin-expressing MDA-MB-468 cells (Fig 6A). The control and asporin-expressing MDA-MB-468 cells were implanted subcutaneously in NOD-SCID mice. Primary tumor growth was monitored weekly. The results indicated that asporin-expressing tumors were significantly smaller, reaching up to 2-fold lower volumes at 7 wk post-engraftment (day 49 post-engraftment: 124.1 mm^3^ smaller than control; 95% CI 75.2–180.4; *p* = 0.001) (Fig 6B). Histological evaluation demonstrated invasive control tumors developing towards the muscle layers, whereas this was not observed in asporin-expressing counterparts (Fig 6C). Further analysis of asporin-expressing tumors evidenced extensive zones of tumor necrosis in the central areas (Fig 6C), as well as numerous cells with condensed chromatin. In the control conditions necrosis was less pronounced, whereas transparent chromatin staining and the presence of nucleoli further characterized tumor cells. The latter suggested a higher proliferation rate in control tumors. The assessment of tumor proliferation based on Ki67 staining showed stronger and more frequent nuclear positivity in the control tumors in comparison to the asporin-expressing counterparts (Fig 6C). IHC evaluation of asporin expression in the experimental tumors evidenced the expected asporin overexpression.

Considering that asporin blocks TGF-β1 activity and EMT, processes known to enrich stem cells, we hypothesized that the abundance of stem cells would be different in these two experimental conditions. The evaluation of tumor stemness, using two different and independent signatures, showed that asporin-expressing tumors had a significantly lower percentage of stem cells (Fig 6D). As CSCs are essential for tumor survival and metastasis, we sought to evaluate tumor dissemination in mice following tumor resection. For this purpose the animal experiments were divided into two separate cohorts: (i) time-matched and (ii) size-matched. For the time-matched cohort, the tumorectomy was performed at week 7. For the size-matched group, the tumorectomy was conducted at week 9 for control and at week 12 for asporin-expressing tumors (Fig 6F). In both instances the mice were allowed to recover and were observed for axial lymph node and lung metastases during an additional period of 3 wk. As indicated by the time-matched data, control mice developed overt lung metastases, whereas this was not observed in the asporin condition (Fig 6E). Control animals consistently developed frequent and large deposits in the lungs. Animals carrying asporin-expressing tumors also showed lung metastases; however, they were less frequent and of smaller size. The notion that asporin is interfering with the process of metastasis was further confirmed in the size-matched experiments. In this cohort the tumor growth was followed for a longer period of time (control mice 9 wk, asporin 12 wk), highlighting an overall 3-wk delay of tumor growth in asporin-expressing mice. The results quantifying metastases 3 wk post-tumorectomy were similar to those of the time-matched condition (Fig 6G). Collectively, the data obtained with both xenograft models suggested that asporin expression inhibits tumor growth as well as metastatic progression.

### High Asporin Levels Delineate Breast Cancer Patients with Good Clinical Outcome

**Fig 7:**
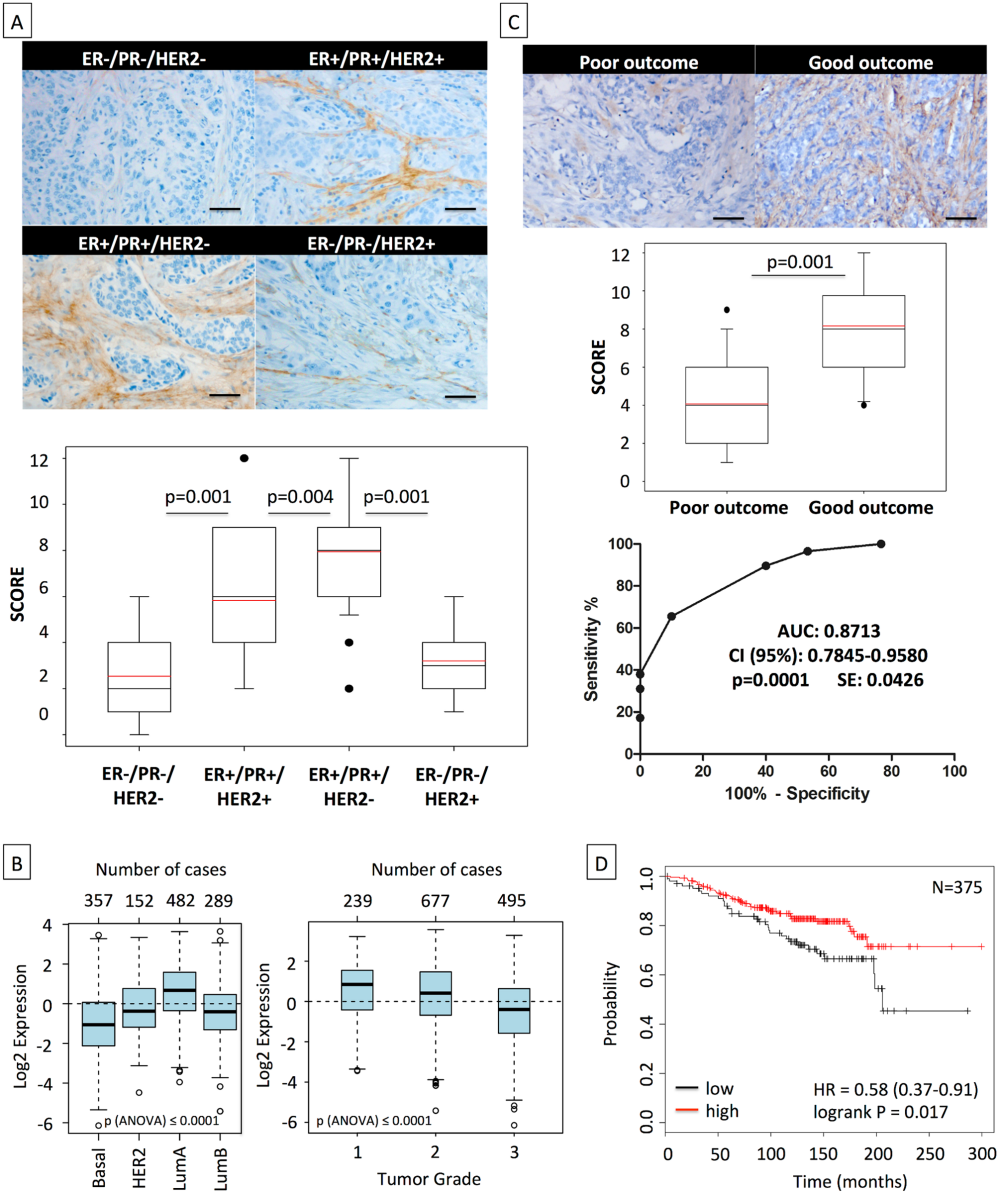
High asporin expression in human breast cancer matches with luminal-like tumor type and good patient outcome. (A) Representative IHC staining of asporin expression in human breast cancer tissues (upper panel). Box plots of asporin expression in 180 breast cancer patients with different status of HER2, ER, and PR are also shown (lower panel). The black line denotes the median expression, and the red line the mean expression. Significant differences in asporin expression were detected among all different subtypes of breast cancer. (B) Analysis of asporin mRNA expression in breast cancer tumors from different molecular subtypes (*n* = 1,280) and evaluation of asporin mRNA expression in breast cancer of different pathological grades (*n* = 1,411). (C) Representative IHC staining of asporin expression (upper panel), box plot showing the IHC score (middle panel) in breast cancer tissues from 60 patients with different outcomes, and ROC curve analysis of data obtained from 60 patients with different outcome (lower panel). Scoring and statistics were performed as outlined in the Methods section. (D) Kaplan-Meier survival curve based on asporin mRNA expression in untreated breast cancer with post-operative follow-up of 25 y (*n* = 375). Images in panels were taken at 100× magnification. All analyses outlined in (B) and (D) were performed using publicly deposited gene expression datasets [31,32] and according to procedures outlined in the Methods. AUC, area under the curve; HR, hazard ratio; SE, standard error.

Considering the in vitro and in vivo data, we expanded our observations using IHC to 180 breast cancer patients, subdivided in four categories with 45 cases each: (i) ER−/PR−/HER2− (triple-negative), (ii) ER+/PR+/HER2+ (triple-positive), (iii) ER+/PR+/HER2− (HR+), and (iv) ER−/PR−/HER2+ (HER2+) (Fig 7A; S1 Table). In our cohort, patients of all subgroups had similar age and showed similar tumor size. Triple-negative and HER2+ cases had higher tumor grade (Bloom 3 versus 2) and a stronger percentage of proliferating cells (Ki67+ cells: ~43% versus ~17%) than the other two subgroups. The frequency of metastasis was highest in TNBC patients (22%), followed by HER2+ and triple-positive breast cancer patients, who displayed similar frequencies (~9%). The IHC results showed that HR+ tumors had a high asporin expression, which was significantly elevated (up to 4-fold) in comparison to TNBC and HER2+ tumors. The latter subgroups had low, and in some cases non-detectable, asporin expression. Intrigued by these findings we aimed to examine the validity of our observations in more individuals. To do so, we used GOBO and publicly deposited mRNA expression data from breast cancer patients [31]. Analysis of asporin mRNA expression in tumors from different molecular subtypes (Fig 7B) confirmed the results obtained with IHC analysis, demonstrating that asporin expression is high in luminal-A and low in basal-like subtypes (*n* = 1,280). Evaluation of asporin mRNA expression in tumors of different pathological grades showed that its expression is higher in grade 1 and lower in grade 3 tumors (*n* = 1,411). This gradual decrease of asporin expression with the grade of the tumor suggested a relationship between asporin expression and breast cancer progression. Thus, we sought to verify how asporin expression correlates with breast cancer patient outcome. We assessed asporin protein expression retrospectively using IHC and tissues from 60 breast cancer patients with over 10-y follow-up (Fig 7C; S2 Table). The patients were divided into two groups: (i) good outcome (signified by no metastatic disease in the follow-up period and following the resection of the primary tumor) and (ii) poor outcome (patients who developed metastases). The two groups showed no major difference in age, tumor size, tumor grade, and ER, PR, HER2, and Ki67 status. The IHC results evidenced significantly higher levels of asporin in patients with good outcome than in patients with poor outcome (2-fold; *p* = 0.001). The suitability of asporin as a biomarker candidate for predicting metastasis in breast cancer patients was evaluated using a ROC curve. As indicated in Fig 7C, the area under the curve was 0.87 (95% CI 0.78–0.96; *p* = 0.0001). These data warranted further examination in a larger cohort of patients, with longer survival follow-up. We therefore examined mRNA expression in breast cancers using Kaplan-Meier Plotter and publicly deposited data [32], where the corresponding patients had a post-operative follow-up of 25 y and had no adjuvant treatment (*n* = 375). The Kaplan-Meier survival curve obtained from these data confirmed the IHC results and demonstrated that low asporin mRNA expression is significantly associated with decreased overall survival (hazard ratio = 0.58; 95% CI 0.37–0.91; logrank *p* = 0.017) (Fig 7D).

## Discussion

A tumor’s ability to successfully grow is increasingly regarded as proportional to the cancer cells’ fitness to survive in a given environment. Their survival is facilitated by adaptation to the environment as well as by actively adapting the environment to the needs of the cancer cell [1,2]. This is in agreement with our key findings that tumor cells with known genetic differences as well as distinct tumorigenic and metastatic potentials have a heterogeneous ability to induce or inhibit asporin expression in stromal fibroblasts. Molecular analysis of all breast cancer cell lines used in the current work [35] suggests that only HR+ cells can induce strong asporin expression in fibroblasts. Contrary to this, CM from TNBC cells strongly inhibits asporin expression in fibroblasts, even when it is induced exogenously by TGF-β1. This, in particular, underlines the evolutionary adaptation of aggressive breast cancer cells. They efficiently exploit a potent cytokine like TGF-β1, yet suppress any unwanted reactions that may result from it (e.g., expression of a natural inhibitor asporin by the stroma). The observations made in vitro were further confirmed in patients, where triple-negative (mainly belonging to basal-like molecular subtype [43]) and HER2+ tumors had the lowest asporin expression. Both are known to be aggressive tumor subtypes with poor clinical outcome [44–46]. The highest asporin levels were observed in the ER+/PR+/HER2− group, which consisted of patients whose tumors were molecularly classified as mainly (~50%) luminal-A subtype [43], which is known to have the best prognosis among all breast tumors [44–46]. Considering this, we were intrigued to identify the mechanism by which TNBC cells manage to suppress asporin expression. Owing to previously published microarray data documenting the differences between triple-negative (e.g., MDA-MB-231) and HR+ (e.g., MCF-7) cells, we identified a strong cluster of several interleukins that were uniquely expressed in TNBC cells. The findings were not surprising, knowing that pro-inflammatory cytokines are essential for “smoldering” inflammation, a key ingredient in cancer and metastasis. The notion that interleukin secretion is responsible for the pro-cancer environment in the tumor stroma context is supported by data from the literature. For example, CXCL1 and CXCL5, secreted by pancreas cancer cells, can activate CXCR2 in fibroblasts to stimulate the production of connective tissue growth factor that, in turn, fuels tumor progression [47]. In the current study we found that IL-1β secreted by MDA-MB-231 cells is indeed responsible for asporin suppression in fibroblasts. The IL-1β protein expression in patient material was inversely correlated with asporin expression. IL-1 consists of two family members, IL-1α and IL-1β, of which only IL-1β is secreted, whereas IL-1α is cytosolic. Earlier studies showed that a single dose of IL-1β is sufficient to significantly increase the number of lung metastases in a melanoma murine tumor model [48]. In line with this, IL-1β expression is elevated in several human tumors (including breast cancer); thus, patients with high IL-1β levels have generally bad clinical outcome [49]. IL-1β induces the expression of several pro-metastatic and pro-angiogenic proteins, among them VEGFA, MMPs, TNFα, and notably TGF-β1 [50]. The present study contributes to further clarifying the intricate mechanism by which IL-1β subverts the tumor stroma into a pro-tumor environment. It does so partly by promoting the expression of tumorigenic cytokines and suppressing their natural inhibitors, in this case asporin, an inhibitor of TGF-β1 [50]. The current findings call for the employment of different strategies to inhibit IL-1β, at least in TNBC. This could be rapidly achieved by employing an anti-IL-1β antibody (canakinumab/Ilaris) or a naturally occurring IL-1β inhibitor IL-1RA (used in vitro in the present work). Recombinant IL-1RA (anakinra/Kineret) is already approved for rheumatoid arthritis treatment, and mounting evidence from gastric [51] and breast cancer [52] research supports its application in treating tumors. A recent phase 1 open-label study with human anti-IL-1 antibody (MABp1) in advanced cancer patients showed encouraging results in terms of disease control, tolerance, and low side effects [53]. Next to considering inhibitors of IL-1β, another axis of treatment could be supported by the asporin peptide (159–205) responsible for TGF-β1 binding. In this work, we showed that the asporin peptide is capable of suppressing different TGF-β1-promoted processes including the acquisition of stem-like phenotype and migration. However, employing the peptide in vivo necessitates further engineering to prevent degradation and increase tissue diffusion. Care would also need to be taken to prevent, or at least diminish, the possibility of an immune reaction against the peptide construct. Future work should certainly address these issues and further explore the possibility of utilizing asporin-derived peptides for the treatment of TNBC, with the aim of slowing progression, suppressing the growth of metastatic lesions, or preventing metastatic dissemination.

Decorin and biglycan are other members of the SLRP family that have been shown to be able to bind TGF-β1 [54,55]. Decorin has been labeled as a “guardian from the matrix” because of its ability to sequester a number of cancer-relevant growth factors [56]. However, what makes asporin unique in this context is that decorin and biglycan are expressed during development and broadly expressed in various normal organs [57,58]. The current study underscores the limited expression of asporin in normal adult tissue, qualifying it also as a target for antibody drug conjugates, and highlights its ability to inhibit TGF-β1 downstream signaling, cancer cell migration, and EMT. The in vivo data outlined here support the idea that asporin acts as a tumor suppressor in breast cancer. Asporin-expressing TNBC cells grow significantly slower and are less invasive when xenografted in mice. Following tumor resection, animals with tumors expressing asporin develop fewer and much smaller metastatic deposits in the lungs. One of the possible explanations for this observation may be the interference of asporin with the process of EMT, which is well known to promote tumor dissemination as well as to induce stem cell phenotype [24]. Indeed, the evaluation of two independent and well-established signatures of CSCs, namely ALDH positivity (general) and the abundance of the CD44^high^/CD24^low^ (breast cancer-specific) population, confirms that asporin-expressing tumors have on average (two signatures together) 50% fewer CSCs. However, recent data on asporin overexpression in gastric cancer show an opposite, pro-invasive function for this stromal protein [17]. For as long as we do not understand all the facets of TGF-β1 biology, the literature may remain a collection of seemingly contradictory findings [59–65]. For example, TGF-β1 inhibition has been previously reported to induce collective cancer cell invasion [66]; hence, it is not surprising that asporin, as a natural inhibitor of TGF-β1, may under certain circumstances contribute to the growth of some tumor types. Therefore, future studies should necessarily take into consideration not only the levels of TGF-β1 but also the expression of its natural inhibitor asporin.

The present retrospective study in breast cancer patients with 10-y follow-up underlines the importance of high asporin expression for good clinical outcome. All results obtained by IHC analysis of protein expression levels are further confirmed by the results generated from publicly deposited gene expression data. Collectively, these findings strongly suggest that asporin should be considered as a future diagnostic and prognostic marker, having the potential to stratify breast cancer patients and identify those who are in need for more clinical attention. Therefore, future prospective studies in more patients are required to evaluate the clinical potential of using asporin as a predictive biomarker in breast cancer.

## Supporting Information

S1 Table Histological characteristics of 180 breast cancer tumors of different subtypes. Values are mean ± SD. Tumor size refers to the diameter (longest axis) of the tumor. Percentage values indicate the proportion of tumor cells that stained positively for the given marker. Frequency of metastasis refers to the respective status at the time point the patient material was collected in the study.
(DOCX)

S2 Table Histological characteristics of 60 breast cancer tumors of patients with 10-y follow-up. Values are mean ± SD. Tumor size refers to the diameter (longest axis) of the tumor. Percentage values indicate the proportion of tumor cells that stained positively for the given marker. Frequency of metastasis and survival refer to the respective status at the end of the follow-up period.
(DOCX)

S1 Text ARRIVE checklist. (PDF)

## Abbreviations

7-AAD: 7-aminoactinomycin D
CAF: cancer-associated fibroblast
CM: conditioned medium
CSC: cancer stem cell
DMEM: Dulbecco’s Modified Eagle’s Medium
EMT: epithelial to mesenchymal transition
ER: estrogen receptor
HR: hormone receptor
IHC: immunohistochemistry
NBF: normal breast fibroblast
PR: progesterone receptor
qRT-PCR: quantitative real-time PCR
ROC: receiver operating characteristic
SD: standard deviation
SEM: standard error of the mean
SLRP: small leucine-rich proteoglycan
TNBC: triple-negative breast cancer
