# Supplementary material for: Asporin Is a Fibroblast-Derived TGF-β1 Inhibitor and a Tumor Suppressor Associated with Good Prognosis in Breast Cancer
Source: PLoS Med. 2015 Sep 1;12(9):e1001871. doi: 10.1371/journal.pmed.1001871 (PMC4556693; doi:10.1371/journal.pmed.1001871)
Supplement: S1 Table — Values are mean ± SD. Tumor size refers to the diameter (longest axis) of the tumor. Percentage values indicate the proportion of tumor cells that stained positively for the given marker. Frequency of metastasis refers to the respective status at the time point the patient material was collected in the study. (DOCX) [file pmed.1001871.s001.docx]

|  | **ER-/PR-/HER2-** | **ER-/PR-/HER2+** | **ER+/PR+/HER2-** | **ER+/PR+/HER2+** |
| --- | --- | --- | --- | --- |
| **Number of patients** | 45 | 45 | 45 | 45 |
| **Age** | 57 ± 14 | 63 ± 15 | 62 ± 12 | 56 ± 12 |
| **Tumor size (mm)** | 27 ± 20 | 29 ± 17 | 19 ±12 | 23 ± 14 |
| **Bloom grade** | 3 | 3 | 2 | 2 |
| **ER (%)** | / | / | 94 ± 10 | 84 ± 19 |
| **PR (%)** | / | / | 65 ± 34 | 49 ± 30 |
| **Ki67 (%)** | 51 ± 28 | 35 ± 18 | 11 ± 9 | 22 ± 17 |
| **Frequency of metastasis (%)** | 22 | 9.1 | 0 | 8.5 |

**S1 Table**
