# Supplementary material for: Asporin Is a Fibroblast-Derived TGF-β1 Inhibitor and a Tumor Suppressor Associated with Good Prognosis in Breast Cancer
Source: PLoS Med. 2015 Sep 1;12(9):e1001871. doi: 10.1371/journal.pmed.1001871 (PMC4556693; doi:10.1371/journal.pmed.1001871)
Supplement: S2 Table — Values are mean ± SD. Tumor size refers to the diameter (longest axis) of the tumor. Percentage values indicate the proportion of tumor cells that stained positively for the given marker. Frequency of metastasis and survival refer to the respective status at the end of the follow-up period. (DOCX) [file pmed.1001871.s002.docx]

|  | **Good Outcome** | **Poor Outcome** |
| --- | --- | --- |
| **Number of patients** | 30 | 30 |
| **Age** | 56 ± 13 | 61 ± 13 |
| **Tumor size (mm)** | 19 ± 12 | 31 ± 21 |
| **Bloom grade** | 2 | 2 |
| **ER (%)** | 59 ± 24 | 78 ± 19 |
| **PR (%)** | 60 ± 26 | 60 ± 26 |
| **HER2 amplification** | 3 out of 30 | 5 out of 30 |
| **Ki67 (%)** | 19 ± 17 | 21 ± 20 |
| **Frequency of metastasis (%)** | 0 | 100 |
| **Survival (%)** | 100 | 54 |

**S2 Table**
